# Rapid switching of vestibulo-motor pathways through voluntary eyelid closure in primates

**DOI:** 10.1038/s42003-025-08956-2

**Published:** 2025-11-20

**Authors:** Emma M. Raat, Omid A. Zobeiri, Jean-Sébastien Blouin, Johannes van der Steen, Kathleen E. Cullen, Patrick A. Forbes

**Affiliations:** 1 Department of Neuroscience, Erasmus MC, University Medical Center Rotterdam, Rotterdam, The Netherlands; 2 Department of Biomedical Engineering, Johns Hopkins University, Baltimore, MD USA; 3 School of Kinesiology, University of British Columbia, Vancouver, BC Canada; 4 Djavad Mowafaghian Centre for Brain Health, University of British Columbia, Vancouver, BC Canada; 5 Institute for Computing, Information and Cognitive Systems, University of British Columbia, Vancouver, BC Canada; 6 Department of Otolaryngology-Head and Neck Surgery, Johns Hopkins University School of Medicine, Baltimore, MD USA; 7 Department of Neuroscience, Johns Hopkins University School of Medicine, Baltimore, MD USA; 8 Kavli Neuroscience Discovery Institute, Johns Hopkins University, Baltimore, MD USA

**Keywords:** Reflexes, Neurophysiology

## Abstract

Understanding how the brain adjusts movement control based on context is key to explaining adaptive behavior. The vestibulo-ocular reflex (VOR), with its well-defined circuitry, demonstrates adaptability—its primary role in stabilizing vision is suppressed when gaze redirection is required but remains resilient even in complete darkness. Here, we investigated VOR responses during intentional eye closure, when the need for visual stabilization is volitionally removed. Using scleral search coils, we measured human VOR responses and found a rapid ~36–90% reduction across movement directions when participants voluntarily closed their eyes, indicating a broad disruption of VOR pathways. This attenuation coincided with eyelid closure and persisted until the eyes reopened. Parallel experiments in monkeys confirmed these findings, ruling out mechanical factors and revealing a conserved neural mechanism across primates. Moreover, attempts to open restrained eyelids increased VOR gains, suggesting that motor commands for eye opening influence vestibular processing even without eyelid movement. These results demonstrate rapid, sustained VOR suppression linked to eyelid motor control, highlighting an energy-efficient neural strategy that dynamically adjusts sensory-motor processing when visual stabilization is certain to be unnecessary.

## Abstract (web summary)

Voluntary eye closure rapidly suppresses the vestibulo-ocular reflex in primates, with suppression lasting until reopening. The effect stems from eyelid motor commands, not visual input, revealing context-dependent reflex gating.

## Introduction

The neural circuits that control movements demonstrate rapid switching based on context to achieve a desired goal. For instance, when gathering information from the environment, the brain stabilizes the visual image on the retina, leading to precise adjustments in oculomotor actions under various contexts. When the goal is to fixate a target while moving the head, the vestibulo-ocular reflex (VOR) is the fundamental neural pathway that contributes to stabilizing the visual field. This vital reflex sends motor commands via a three-neuron arc to move the eyes in the direction opposite to head motion. However, if the goal is to redirect gaze to a new target during head motion, the VOR would be counterproductive as it would rotate the eye in the opposite direction of the intended change in gaze. Indeed, under this context of redirecting rather than stabilizing gaze, the VOR is dynamically inhibited^1–5^—effectively switched off—via brainstem premotor saccadic and pursuit pathways^6^.

The sensorimotor circuits that control the VOR are also modulated over longer time frames by changes in sensory feedback. This modulation is essential for the adaptation required to ensure behavioral precision. Notably, visually-induced changes in VOR efficacy are driven when the relationship between head movements and the resulting visual feedback is altered. Numerous studies, using prism glasses or moving visual fields, have established how visual feedback produces changes in the amplitude and timing of VOR eye movements account for these new sensorimotor relationships (see ref. ^7^). Climbing fibers in the cerebellum encode a visual error signal, which induces learning in Purkinje cells and subsequent modulation of intermediate neurons of the VOR in the vestibular nuclei (see ref. ^8,9^). Surprisingly, however, in complete darkness, where visual feedback is entirely absent, VOR-driven corrective eye movements continue to persist. Perhaps even more impressive is the observation that patients with acquired blindness continue to produce VOR response even after two decades without vision^10,11^. This resilience of the VOR suggests that a baseline level of responsiveness can be maintained over the long term, even in a context where the immediate task of stabilizing the visual field seems explicitly irrelevant.

While these previous experiments have demonstrated the VOR’s remarkable resilience to the removal of extrinsic visual feedback, it remains unknown whether its efficacy is dynamically modulated when the eyes are intentionally closed, voluntarily eliminating the need to stabilize the visual scene on the retina. Despite studies reporting conflicting effects of eye closure on the VOR^12–15^, the prevailing view is that closing the eyes has no inhibitory effect on VOR behavior. This interpretation aligns with the persistence of the VOR in complete darkness and acquired blindness but is at odds with principles of energetic optimization in sensorimotor systems^16–20^, given that eye movements are functionally irrelevant for visual acuity when the eyes are voluntarily closed. Importantly, related evidence from another reflexive oculomotor behavior—the torsional optokinetic nystagmus—has shown that reflexive eye movements can be actively suppressed during blinks^21^. Accordingly, here we directly investigated whether the VOR shows context-dependent switching, transitioning from an effective compensatory response to an attenuated state during volitional and sustained eyelid closure. We recorded eye movements using scleral search coils during predictable and unpredictable whole-body rotations with eyes open or closed in complete darkness. Remarkably, voluntary eyelid closure suppressed VOR by up to 90%, with the reduction precisely coinciding with closure and lasting until reopening. This effect was confirmed in nonhuman primates, ruling out mechanical artifacts and demonstrating a conserved mechanism across primate species. Finally, when participants attempted to open their eyelids against an external restraint, compensatory VOR responses partially returned, suggesting motor commands influence vestibular processing independent of eyelid movement. Together our results establish that voluntary eyelid closure attenuates the VOR via eyelid motor pathways, thereby minimizing energetic costs when visual stabilization is unnecessary.

## Results

### Eye closure attenuates VOR gains during sinusoidal motion in all directions

**Fig. 1:**
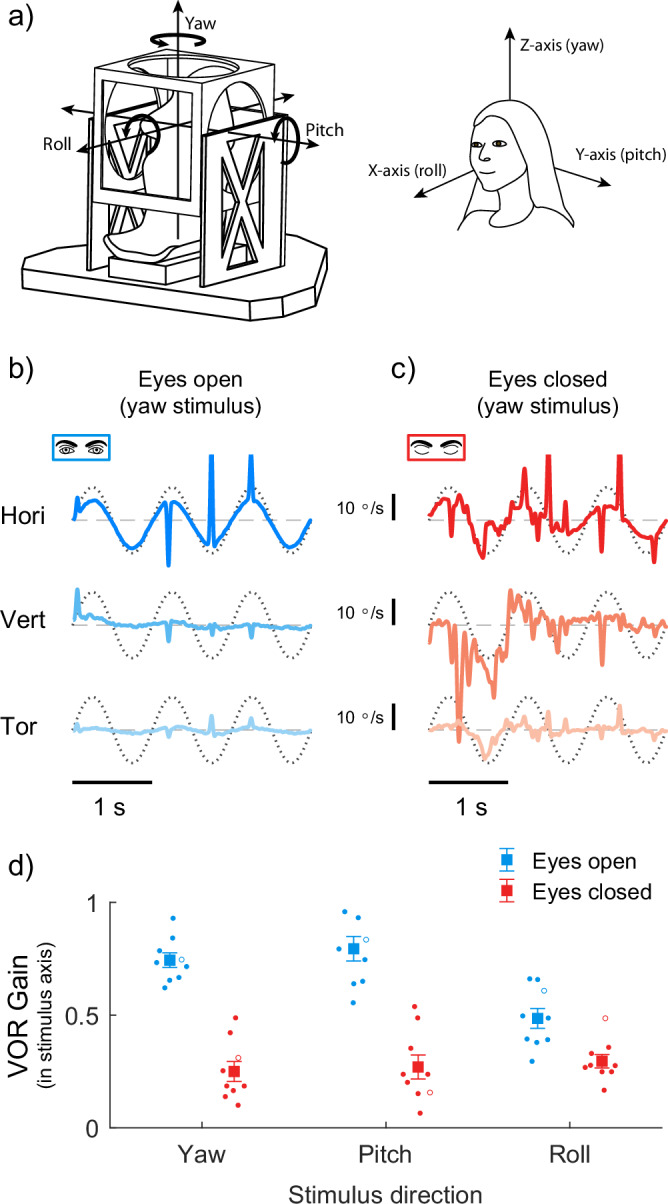
Human vestibulo-ocular reflexes during whole-body sinusoidal rotations with the eyes open and closed. **a** We used a six-degree-of-freedom motion platform to induce whole-body rotations along the three cardinal axes, yaw, pitch, and roll, to evoke horizontal, vertical, and torsional VOR eye movements, respectively. **b** When the eyes were open, compensatory horizontal VOR responses were observed during whole-body yaw motion at 1 Hz (amplitude 12 °/s). VOR responses in the vertical and torsional directions were absent as expected. **c** When the eyes were closed, VOR responses were diminished and did not effectively compensate for the applied motion. VOR responses in the vertical and torsional direction were absent. **d** Group average (large symbols) and individual participant (*n* = 9) VOR gains estimated from yaw, pitch, and roll rotations with the eyes open and closed. The representative participants depicted in (**b**, **c**) are shown as open circles in each stimulus direction for the eyes open and closed conditions.

To directly investigate whether the VOR shows context-dependent switching from an effective compensatory response to an attenuated state during eyelid closure, we recorded eye movements evoked in human participants in response to externally applied head movements. All experiments were completed in complete darkness, with participants comfortably seated on a motion platform or a stationary chair. In our first experiment, we examined eye movement responses evoked by low-frequency (1 Hz) sinusoidal head motion resulting from whole-body movements about each of the three cardinal axes (roll, pitch, and yaw; see Fig. 1a). Data from a representative participant during whole-body movement in the yaw axis are shown in Fig. 1b, c. When this participant had their eyes open, eye movements were observed primarily in the horizontal direction, in the direction opposite to the applied whole-body yaw motion consistent with a compensatory VOR response (gain = 0.96). In contrast, when the participant closed their eyelids, eye movements became more oscillatory (i.e., noisy) and VOR compensation to the applied sinusoidal yaw motion was disrupted, exhibiting much smaller gains (~0.21). The noisy eye movements observed were characterised by small oscillations (~5–15 °/s) in all directions, and were similar to those reported in previous studies during prolonged eyelid closure in stationary participants (see ref. ^22^). Notably, in our experiment, these oscillations were superimposed on the reduced VOR-driven eye movements evoked by the applied whole-body motion.

At the population level, our group data confirmed the dependence of VOR responses on the state of eye opening in all directions of the applied motion. While participants (*n* = 9) had their eyes open, VOR gains (Fig. 1d) during motion about the yaw, pitch, and roll axes were 0.74 ± 0.03, 0.79 ± 0.05, and 0.49 ± 0.04, respectively (main effect of direction: F_2,16_ = 5.313, *P* = 0.017). Furthermore, as expected, in the eyes open condition, these axis-dependent responses, which were characterized by lower gains in roll and higher gains in yaw and pitch, aligned with the direction-dependent nature of VOR (see also refs. ^23–27^). However, when participants closed their eyes, VOR gains along the yaw, pitch and roll axes markedly decreased to 0.25 ± 0.04, 0.27 ± 0.05 and 0.30 ± 0.03, respectively (main effect of eye closure: F_1,8_ = 79.075, *P* < 0.001). We also observed, a significant interaction between movement direction and eye closure (F = 13.333, *P* < 0.001). Pairwise comparisons of the relative change in VOR along the cardinal axes (i.e., roll, pitch and yaw) revealed that eye closure induced a greater decrease in VOR gains during motion along the yaw and pitch (67 ± 6% vs. 63 ± 9%; *t*_8_ = 0.3, *p* = 0.768) axes as compared to the roll axis (36 ± 8%; both *t*_8_ > 2.9, *p* < 0.05). Finally, correlation analysis revealed no significant relationship between VOR gains in the eyes-open and eyes-closed conditions for each movement direction (all *p* > 0.05), indicating that individual differences in the eyes-open state do not reliably predict gains when the eyes were closed. Taken together, these results revealed a significant 36–67% reduction in VOR gains during eye closure in humans across all axes during sinusoidal whole-body rotation.

### Eye closure attenuates VOR gains during unpredictable movement

**Fig. 2:**
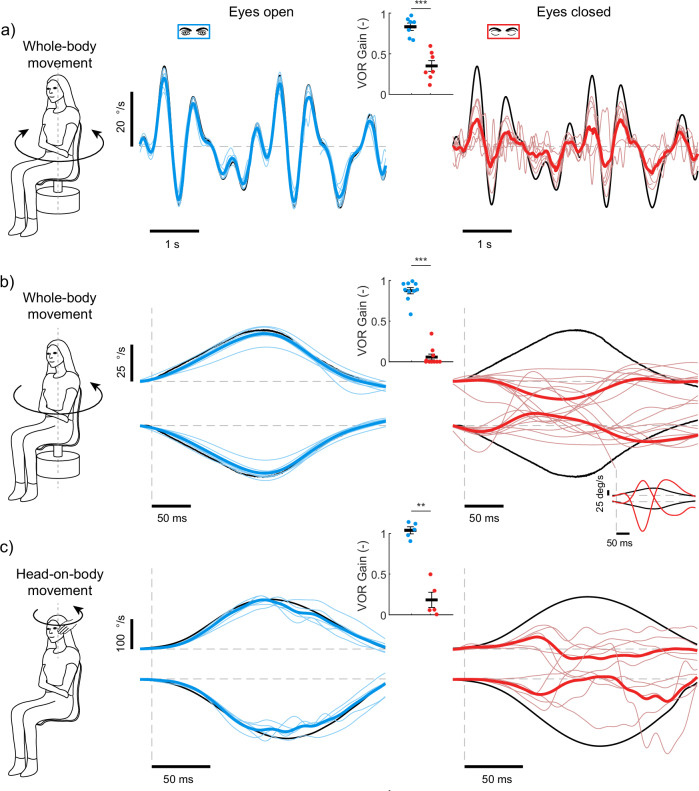
Human vestibulo-ocular reflexes during unpredictable continuous and transient whole-body and head rotations with the eyes open and closed. **a** VOR responses during unpredictable whole-body yaw rotations delivered as a pseudorandom (i.e., sum-of-sines) motion. **b** VOR responses with the eyes open and closed during transient whole-body yaw rotations. Whole-body motion was applied as an acceleration step (280 °/s^2^) over a period of 125 ms and achieved a peak velocity of 35 °/s. **c** VOR responses with the eyes open and closed during transient head-on-body rotations. These experimenter-applied head rotations approached ~150 °/s applied over a period of ~100 ms. In all conditions, VOR gains (see insets in all subpanels) significantly decreased when the eyes were closed; *** indicates *p* < 0.001 and ** indicates *p* < 0.01. Thick black lines and error bars in the insets indicate the group averages and SEMs for each condition (*n* = 7, *n* = 10 and *n* = 5). Notably, eye closure during transient whole-body rotations and head-on-body rotations (**b**, **c**) induced more substantial decreases in VOR gain (~93 and 79%, respectively) as compared to the pseudorandom whole-body rotations (**a**, ~58%). In all conditions (**a**–**c**), thin lines represent individual participant responses while thick lines represent the group average.

In the previous section, we examined VOR gains during continuous low-frequency sinusoidal head movements, which are predictable due to their cyclic nature. As a result, we next investigated the effect of eye closure on VOR gains using continuous pseudorandom and transient unpredictable whole-body yaw movements (see Methods). First, we recorded eye movements in a group of participants (*n* = 7) during continuous pseudorandom (i.e., sum-of-sines) whole-body rotations (max velocity 30 °/s) in the yaw-direction (see Fig. 2a). During these trials, participants either kept their eyes open or closed throughout. In the eyes open condition, VOR gains ranged from 0.8 to 0.95 (0.83 ± 0.05), indicating robust compensatory eye movements, similar to those observed in sinusoidal movements. However, when participants closed their eyes, VOR gains decreased by ~58% (0.35 ± 0.07, *t*_6_ = 6.21, *P* < 0.001). These findings demonstrate that eye closure markedly reduces VOR evoked by unpredictable as well as predictable continuous stimulation.

We next explored whether comparable suppression of the VOR occurs when the applied head motion is an unexpected, transient event rather than a continuous stimulus. Two groups of participants were exposed to brief (~100–125 msec) transient vestibular stimuli: whole-body accelerations (*n* = 10; max velocity 35 °/s) or head-on-body rotations (i.e., the head impulse test; *n* = 5; max velocity ~150 °/s). With the eyes open, compensatory eye movements were observed in the opposite direction to the head movement, resulting in VOR gains of 0.87 ± 0.04 and 0.88 ± 0.04 for whole-body and head-on-body rotations, respectively. However, when equivalent head movements were applied with the eyes closed, the eye movements deviated substantially from the onset of the applied motion, showing little or no compensation (see Fig. 2b, c). Instead, in many cases the primary eye rotations were anti-compensatory, moving in the same direction as the whole-body motion (7 of 10 for whole-body and 3 of 5 for head-on-body rotations). In one participant (see Fig. 2b inset), the velocity of these anti-compensatory eye movements was four times greater than the applied motion. We therefore quantified only compensatory responses, assigning a value of zero to negative gains from anti-compensatory responses. This resulted in decreases of approximately 93% and 79% for our estimations of VOR gain in the eyes closed condition during whole-body and head-on-body stimuli, respectively (eyes closed VOR gains: 0.06 ± 0.04, *t*_9_ = 14.5, *P* < 0.001 and 0.18 ± 0.09, *t*_4_ = 7.36, *P* = 0.006). Overall, these results show that closing the eyes prior to transient unpredictable head movements suppresses the VOR from the onset of the applied motion.

### Eye closure rapidly attenuates the VOR across species

**Fig. 3:**
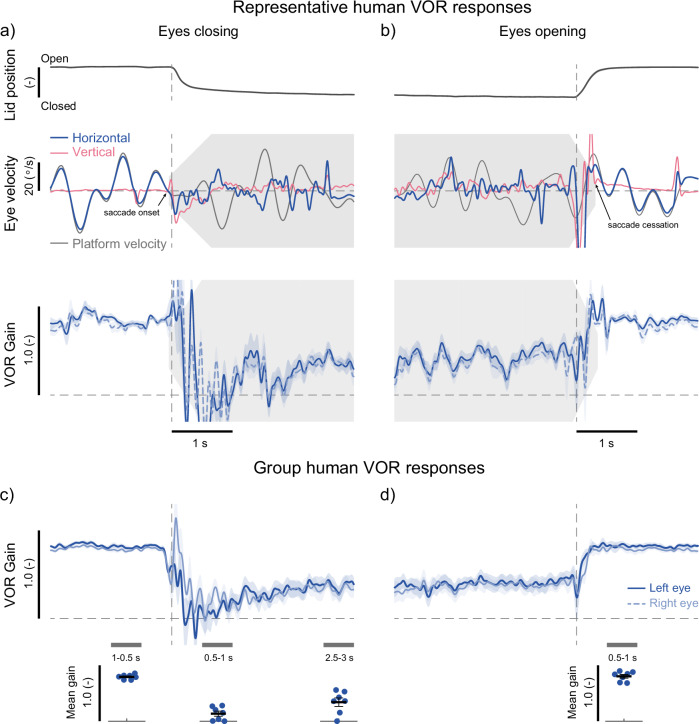
Human vestibulo-ocular reflexes throughout the time course of eyelid closure while participants were exposed to continuous whole-body yaw rotations. **a** Eyelid position (top), eye velocities (middle), and average VOR gain (bottom) traces during eye closure from a representative participant. The eyelid position trace is the average over the 20 repetitions; the derivative of eyelid traces was used to identify time zero in the traces (see Methods). In the middle plot, platform velocity (black traces) is shown together with horizontal (blue traces) and vertical (pink traces) eye velocities from the left eye during a single trial of eyelid closure. Saccade onset at eyelid closure and saccade cessation at eyelid opening are identified in the example traces with small arrows. VOR gain traces in the bottom plot are averaged over the 20 repetitions of eye closure for each eye. The solid line represents the average VOR gain of the right eye, while the dotted line represents the average VOR gain of the left eye. The shaded regions represent the SEM across repetitions (*n* = 20). **b** Equivalent responses (eyelid, eye velocity, and VOR gain) of the same representative participant as those depicted in (**a**), but during eye opening. **c**, **d** Group-averaged VOR gains (*n* = 7) during eye closing and opening, respectively. The shaded regions represent the SEMs across participants (*n* = 7). Insets below the traces depict the mean VOR gain per participant over the time periods indicated by the thick grey bars pre- (1–0.5 s) and post-eyelid closure (0.5–1 s and 2.5–3 s), and post (0.5–1 s) eyelid opening. The individual participant responses are the average of both eyes. Thick black lines and error bars indicate the group average and SEM (*n* = 7). Tapered grey areas in all plots indicate when the vertical saccades were estimated to start and end during eye closing and opening (see Methods).

So far, we have shown that the VOR gain decreases when the eyes are closed prior to the onset of continuous and transient head movements. Next, we directly established the time course over which eye closure influences VOR gain. Participants (*n* = 7) were instructed to voluntarily close and open their eyes during continuous pseudorandom whole-body yaw-rotation. Figure 3a, b illustrate data from a representative participant during eye closing and opening, respectively, including: (i) eyelid position, (ii) horizontal and vertical eye velocities, and (iii) the average VOR gain estimates computed across all repetitions.

With the eyes open (prior to eye closure), the participant made horizontal eye movements that compensated for whole-body motion (Fig. 3a), while vertical eye movements remained minimal. The average horizontal VOR gain (averaged across eyes and repetitions) over the period of 1–0.5 s before eye closure was 0.79 ± 0.02. Just before and during eyelid closure (starting at *t* = 0), brief transient eye movements occurred in both the horizontal and vertical directions with velocities exceeding ~50 °/s. These movements included a transient change in eye velocity that began 49 ± 19 ms before eyelid motion that ended (i.e., velocity approaching zero) 492 ± 168 ms after it started (see Methods). These eye movements led to erratic fluctuations in VOR gains, briefly exceeding 1 and dropping below 0 throughout eyelid closure. As a result, the average VOR gain in the 0.5–1 s window after eye closure was near zero (0.12 ± 0.04). By 2.5–3 seconds post-closure, the gain increased and stabilized at 0.37 ± 0.04 (see Fig. 3a, b). During this time, the eyes underwent slow tonic deviations in horizontal and vertical eye position that occurred from 1 to 2 s post closure and were accompanied by low-velocity oscillations (~10–15 °/s) in both horizontal and vertical eye movements. Note that the small oscillations were similar to those observed during sinusoidal rotations (see Fig. 1c). As noted above, these transient, tonic, and oscillatory movements at eye closure are consistent with previous observations in stationary participants^22^. Finally, upon eye opening, transient horizontal and vertical eye movements reappeared, starting 64 ± 57 ms before eyelid motion and ending 349 ± 196 ms after eyelid motion began. Compensatory eye movements resumed when the lids fully opened, with a VOR gain of 0.85 ± 0.02 in the 0.5–1 s window after eye opening.

The rapid modulation of VOR gains that accompanied the onset of eye closure was confirmed in the group data (Fig. 3c, d). Transient horizontal and vertical eye movements, on average, began 56 ± 24 ms prior to the onset of eyelid motion (i.e., *t *= 0 s) and ended 441 ± 42 ms after eyelid motion onset. In addition, coincident with the onset of these transient eye movements, estimated VOR gains dropped to near zero values, compared to an average of 0.79 ± 0.02 with the eyes open (i.e., *t* = 1-0.5 s before closing) (see Fig. 3c). Notably, the initial decrease in VOR gains occurred prior to the transient eye movements, likely due to the 150 ms window used for estimation.

Following complete closure of the eyes (0.5–1 s after eyelid closing), VOR gains initially dropped to 0.13 ± 0.05 but then stabilized at 0.34 ± 0.08 within 2.5–3 seconds. A repeated measure ANOVA revealed a significant change across all three time periods (F_2,12_ = 56.3, *p* < 0.001). Pairwise comparisons indicated significant differences between the periods just before and after eye closure (both *p* < 0.003) but no significant change between the two periods after closure (*p* = 0.060). When participants then opened their eyes, rapid eye movements were again observed starting 108 ± 44 ms prior to eyelid movement and then ending 251 ± 79 ms after eyelid movement, causing erratic changes in VOR gains (see Fig. 3d). However, gains then returned rapidly to pre-closure levels of 0.80 ± 0.03 by the time the eyelid was fully raised (i.e., 0.5–1 s after eyelid opening). These results indicate that eye closure induces rapid and sustained attenuation of the VOR that initiates prior to detectable changes in eyelid movement, and that the VOR gain then returns to expected levels over a similar time course (within 1 second) once the eyes are again opened.

**Fig. 4:**
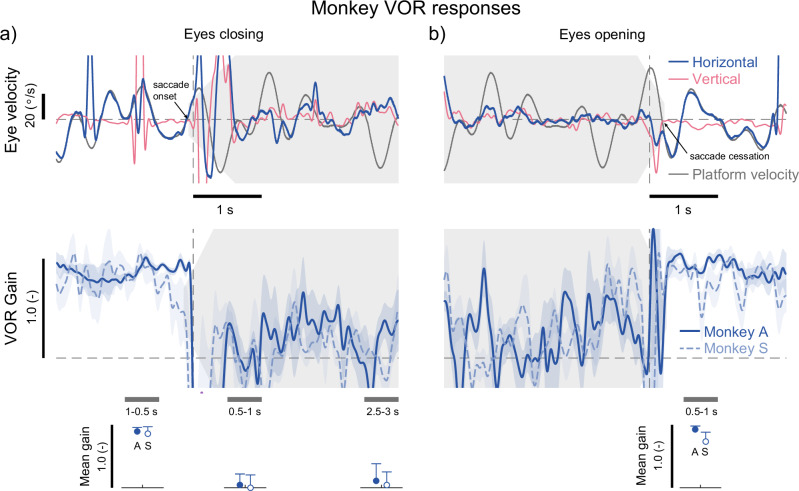
Monkey (*n* = 2) vestibulo-ocular reflexes throughout the time course of eyelid closure while the animals were exposed to continuous whole-body yaw rotations. **a** Horizontal and vertical eye velocities (top) and VOR gains (bottom) traces during eye closure. Eye velocities from the right eye are shown during a single occurrence of eye closure. Saccade onset at eyelid closure and saccade cessation at eyelid opening are identified in the example traces with small arrows. Time-varying VOR gains are the average of all occurrences of eye closure collected from each of the two monkeys; lines and shaded regions represent the mean and SEMs across repetitions (*n* = 15), respectively. Time zero was identified by the first peak in vertical eye velocity around the time of the experimenter-identified eyelid closure region. Insets below the lower plot depict the mean VOR gain responses for each monkey over the time periods indicated by the thick grey bars, including: pre- (1–0.5 s) and post-eyelid closure (0.5–1 s and 2.5–3 s). Error bars indicate the SEM across repetitions (*n* = 15) within each animal and for clarity are depicted here only above the mean. **b** Equivalent responses as those depicted in (**a**), but during eye opening. Inset below the lower plot depicts the mean VOR gain responses for each monkey over the time period post-eyelid opening (0.5–1 s). Tapered grey areas in all plots indicate when the vertical saccades were estimated to start and end during eye closing and opening.

One potential explanation for our findings so far is that during eyelid closure, the restriction or slippage of the coil relative to the eye, rather than an actual change in efficacy of vestibular-drive VOR eye movements, caused the apparent change in VOR gain. To test this possibility, we replicated the experiment in a non-human primate model to ensure that mechanical restriction or slippage of the scleral coil was not responsible for the modulation of the VOR in humans. In the primate model, the coil was surgically implanted in the sclera, ensuring it remained fixed for all eye movements, unlike in human participants where it was mounted in a contact lens. As shown in Fig. 4, eyelid closure in monkeys resulted in eye movement responses and VOR gain modulation with features comparable to those observed above in our human participants. First, transient eye movements in both horizontal and vertical directions were observed during eyelid closing and opening (Fig. 4a, upper traces). Second, VOR gains rapidly decreased from ~0.88 (monkey A: 0.90; monkey S: 0.86) with the eyes open 1–0.5 s before closing to ~0.03 (monkey A: 0.05; monkey S: 0.01) 0.5–1 s after the eyes were closed, slightly increasing to ~0.07 (monkey A: 0.11; monkey S: 0.04) after 2.5–3 s (see Fig. 4a, lower traces). Although animal VOR gains over the 2.5–3 s after eyelid closure were on average lower than VOR gains in humans, there are several intervals ~1–2.5 s after eyelid closure where the gains matched our human data. Finally, VOR gains then returned to expected levels of approximately 0.84 (monkey A: 0.93; monkey S: 0.74) 0.5–1 s after the eyes were opened (see Fig. 4b, lower traces). These changes in VOR gains based on eye closure closely mirrored those seen in human experiments, supporting the interpretation that the modulation of human VOR is driven by neural modulation rather than mechanical restriction caused by the scleral coil.

### Motor signals of eye closure drive the suppression of VOR

**Fig. 5:**
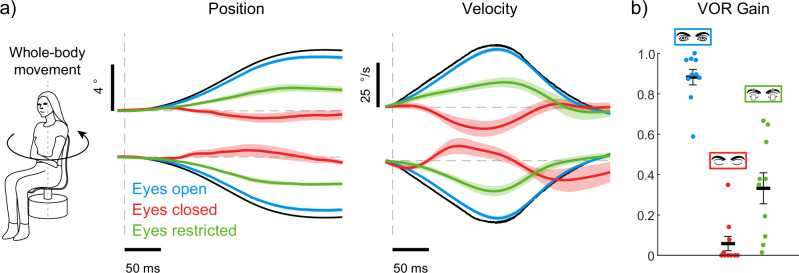
Partial vestibulo-ocular response recovery with the eyes restricted closed while participants attempted to open the eyelids. **a** VOR position and velocity responses to the transient whole-body motion (black line). For the eyes restricted condition, participants were instructed to attempt to open their eyes when an audio cue was given ~4 seconds before the motion was applied. Participants from this experiment also performed the eyes open and closed conditions that were reported in Experiment 2. Therefore, data from the eyes open and closed conditions are replicated from Fig. 2 for comparison. The solid lines represent the average position and velocity responses and the shaded regions represent the SEM across participants (*n* = 10). **b** Average VOR gains for eyes open, eyes closed, and eyes restricted conditions. When participants attempted to open their eyes, VOR gains increased to intermediate levels (~0.35) but did not reach values observed with the eyes open (~0.87). Thick black lines and error bars indicate the group average and SEM (*n* = 10).

Finally, to determine whether the modulation of the VOR relies on the motor commands generated to open or close the eyelids, we externally imposed a prolonged eyelid restraint on participants (see Methods) while they experienced unexpected transient whole-body yaw motion. Approximately 3–4 seconds prior to each movement, an audio cue instructed participants to attempt to open their eyes against the physical restraint. The average group data for eye position and velocity under these conditions are shown in Fig. 5. Note, that these conditions were tested in participants who also performed the eyes open and closed conditions during transient whole-body movement trials in Experiment 2; therefore, these responses are replicated here from Fig. 2. Comparison of these trials to recordings of eye movements when the eyes were naturally closed reveal the return of compensatory eye movements in the direction opposite to the applied motion (see Fig. 5). Notably, average VOR gains significantly increased from 0.06 ± 0.04 to 0.33 ± 0.08 (*t*_9_ = 3.5, *p* = 0.006). This result suggests that the motor commands to open the eyelids actively engaged the VOR, enabling it to appropriately compensate for ongoing head movements. However, VOR gains did not return to the same level observed during eyes open conditions (0.87 ± 0.04), suggesting that a motor-dependent engagement of the VOR may not be the only factor contributing to this modulation.

## Discussion

In this study, we demonstrate that the VOR—an essential neural mechanism for stabilizing vision through compensatory eye movements during head motion—is suppressed during voluntary eye closure when gaze stabilization is unnecessary. This scenario contrasts with conditions such as complete darkness or acquired blindness, where visual feedback is externally removed, and the VOR remains robust despite its diminished role in visual stabilization. We first compared the efficacy of the VOR responses evoked by head motion stimuli with the eyes open versus eyes closed in darkness and consistently found a marked attenuation (~36–90% reduction) during eye closure. This active attenuation occurred for head motion about the yaw, pitch, and roll axes, indicating a general reduction in the VOR pathway efficacy in all three dimensions. Our analysis further revealed that the timing of this attenuation was tightly linked to eyelid closure. VOR gains dropped precipitously at eyelid closure (~84% reduction) and then stabilized to ~0.3 within ~1–3 seconds. Correspondingly, we found VOR gains then returned to expected levels (~0.8) within <1 second of participants opening their eyes. Parallel experiments in a non-human primate model confirmed these observations, ruling out mechanical factors related to scleral coil restriction. Finally, VOR gains were partially restored when participants attempted to open their eyelids against a physical restraint. Thus, taken together, these results establish that robust attenuation of the VOR occurs with voluntary eye closure, likely reflecting context-dependent modulation via eyelid motor control pathways that inhibit the VOR to reduce energetic expenditure when it is not functionally necessary.

Our central finding that VOR efficacy decreases during voluntary eye closure contrasts with previous studies that have shown intact VOR responses to applied head movements^12,13^. We propose that methodological differences may explain the contradicting outcomes. First, a technical limitation of Weissman et al.^13^ was the use of EOG. We speculate that the use of EOG introduced artifacts during eyelid closure, as EOG recordings with the eyes closed can lead to inaccurate vertical (up to 50° off) and horizontal (including oppositely directed) eye movements. This occurs because the eyelid functions as a sliding electrode, shunting the positive corneal pole with the EOG electrodes^22^. Indeed, earlier studies using EOG to access the status of the VOR with eyes closed during head rotations have provided mixed results, with some studies reporting decreases^14,15^ and others reporting no change as noted above^13^. Correspondingly, another technical limitation of Shaikh et al.^12^ may stem from their reliance on peak eye movement responses over a restricted observation period to estimate VOR gains. Here, we calculated VOR gains by performing a linear regression between eye and head velocity during the first 100 ms of the acceleration phase. This approach explicitly targeted the compensatory VOR throughout the movement, excluding saccades from the analysis.

Overall, our eye movement recordings using search coils in humans across various head movement protocols (e.g., sinusoidal, sum of sines, and unexpected transients) revealed that voluntary eye closure disrupts the VOR across all directions of head movement. Although blink-related torsional slips can occur when coil leads exit nasally^28^, such artefacts are millisecond-long and <3° in amplitude. By contrast, our ~36–90% drop in VOR gain persisted for the full eye-closed period and affected horizontal, vertical and torsional components. Furthermore, a similar VOR suppression was observed with implanted coils in monkeys, ruling out mechanical factors related to interactions between the eye search coil and the eyelid. Importantly, we note that we also controlled our human participants’ arousal during our procedures—an additional confound of VOR responses proposed by Weissman et al.^13^—by requiring them to open or close their eyes between every trial (~10–15 s) or in response to a sound cue. The similarity in the VOR suppression observed between humans and the non-human primate model strongly suggests that this response is a conserved mechanism across species. A similar blink-related suppression has been reported for torsional optokinetic nystagmus^21^, indicating that eyelid motor commands can down-regulate diverse reflexive eye-movement pathways whenever visual stabilization is unnecessary.

By tracking vestibular-induced eye movements during continuous head motion, we further revealed precise and immediate coupling between the onset of VOR suppression and voluntary eye closure. VOR gains decreased just prior to eyelid movement (~50 ms, Fig. 3) and approached zero within the first second of eye closure. The initial suppression of the VOR further coincided with the generation of transient eye movements in both the horizontal and vertical directions; similar eye movements have also been observed during voluntary eye closure and blink-associated eye movements (BAEMs) reported in stationary participants^29^. The tight alignment of eye closure with both transient eye movements and the initial decrease in VOR gains in both humans and monkeys provides insight into the neural mechanisms underlying this rapid VOR suppression. Notably, following spontaneous and induced blinks, pontine omnipause neurons—which gate the premotor burst neurons responsible for generating saccadic eye movements (reviewed in Cullen 2023^7^)—are rapidly inhibited within 10 ms^30–34^. Crucially, however, Schultz et al.^33^ showed that BAEMs start ~10 ms before the inhibition of the omnipause neurons that are responsible for normal saccadic eye movements, suggesting that these transients are generated by eyelid-related circuitry. Moreover, the OPN pause lasts only ~50 ms^33^, whereas our VOR gain remains depressed for several seconds; thus, a transient release of the saccadic system is unlikely to sustain the prolonged suppression. We therefore propose that voluntary eyelid closure suppresses the VOR through longer-lasting pre-motor inhibition of vestibular pathways, with saccadic-network involvement being secondary.

Interestingly, we found that following the initial VOR disruption at eye closure, the efficacy of the VOR gradually increased to ~0.3 over a 1–3 second period. This sustained suppression was then maintained for up to 60 seconds during both continuous sinusoidal and pseudorandom testing (Exp 1 and 2). Together, these observations indicate that transient eyelid closure initiates a reduction in VOR efficacy that is then maintained until eyelid opening (Exp 3). This sustained reduction in VOR gain suggests that a tonic inhibitory drive, likely based on the integration of the initial eyelid closure motor command, reduces the VOR efficacy throughout the duration of eyelid closure. The regulation of the VOR by eyelid motor commands is further supported by an increase in VOR gain when participants attempted to open their eyes under eyelid restriction, compared to when their eyes were naturally closed (Fig. 5). This VOR gain recovery, however, was partial, suggesting that sensory feedback of the eyelids being closed or feedback interaction with the motor command to open the eyes (i.e., sensorimotor mismatch) may also modulate VOR responses. Importantly, our experiments were conducted in complete darkness, making luminance driven retinal contributions to the observed gain suppression unlikely^35,36^. Collectively, our findings strongly support sustained as well as dynamic interactions between the motor command for eyelid closure and the VOR. Thus, building on the connections between blink-related eye closures and transient changes in eye movements discussed above, we speculate that the inhibitory drive through eyelid motor commands accompanying voluntary eye closure may also engage the premotor ocular pathways during prolonged eye closure. However, further experiments are necessary to elucidate the precise neural mechanisms underlying these sustained changes in VOR efficacy during eye closing and opening.

In this context, it is further noteworthy that the suppression of VOR gains induced by eye closure was more pronounced for transient head movements than continuous stimuli. In addition, 60–70% of participants showed anti-compensatory eye movements in response to transient head motion with their eyes closed. We propose that the more robust VOR suppression and the presence of anti-compensatory eye movements for transient head motion are due to the variability of the evoked ocular responses and the shorter duration of the transient head motion. Indeed, the lower frequency components and the necessary averaging associated with extracting VOR gains in response to continuous head motion filtered the reported responses, thus attenuating or removing the effects of anti-compensatory VOR responses. A careful examination of the ocular movements during the continuous head perturbations reveals brief periods of anti-compensatory responses (see Figs. 1 and  3). Given the proposed interactions between eyelid motor commands and premotor ocular pathways, we speculate that maintaining the eyes closed leaves the superior colliculus-omnipause-burst neuron networks in a variable state. However, further experiments are needed to confirm this possibility during sustained eyelid closure. The resulting variability in this network would lead to variable eye movements, which would have a greater influence on VOR gains estimated during transient head movements.

The efficacy of the VOR is also rapidly reduced in another behavior—specifically, when the goal is to redirect gaze to a new target using combined eye-head movements, or gaze shifts^1–5^. During gaze-shifts, a fully intact VOR would not only be irrelevant but it would actually be counterproductive as it would rotate the eye in the opposite direction to the intended change in gaze. It has been well demonstrated that the VOR is effectively switched off during gaze shifts via the brainstem premotor saccadic pathway^6^. A main difference between gaze shift- and eye closure-related VOR inhibition would be that during gaze shifts, the primary modulation of the premotor pathways arises directly from activity in the superior colliculus. In contrast, we propose that the eye closure-related VOR inhibition observed in the present study is mediated via reciprocal interactions between eyelid closure pathways and the brainstem premotor saccadic pathway (e.g., omnipause neurons), similar to what has been described for blink-related mechanisms as mentioned above^34,37^. One benefit associated with the sustained suppression of the VOR during eye closure may be a reduction in energetic expenditure when it is not functionally relevant to stabilize gaze. Transforming head motion into compensatory eye motion incurs metabolic cost, involving both sensory-motor neural processing and the activation of the oculomotor muscles that move the eyes^17–20^. Although energy optimization in sensory systems is primarily observed when the need for high sensory acuity is diminished (on both short and long time-scales)^20^, recent work on locomotion and arm movements suggests humans may continuously and rapidly adapt behaviour to optimize energy use^38,39^. However, we do not suggest that VOR suppression eliminates all oculomotor energy expenditure during eye closure. Transient high-velocity eye movements that occur while the eyes are closed likely carry their own metabolic cost, and reacquiring fixation after lid opening may also impose a brief additional demand. These factors may limit the net energetic savings. Overall our findings suggest that energy conservation may be one contributing factor to the downregulation of the VOR. This task-dependent modulation minimizes unnecessary sensory processing and motor output when visual stabilization is irrelevant, consistent with prior evidence showing that VOR gain is suppressed during purposeful gaze shift^6,40^.

In conclusion, our study shows a significant decrease in VOR gains during eye closure, revealing context-dependent switching that suppresses the effectiveness of this essential reflex pathway. Furthermore, this change occurs rapidly and stabilizes at a reduced efficacy within seconds, suggesting an energy conserving adaptive response when visual stabilization is unnecessary. The timing of this robust attenuation of VOR was tightly linked to eyelid closure, consistent with modulating mechanisms that are mediated by eyelid motor control pathways. Future studies should aim to investigate the exact neural circuits involved, and examine how these findings translate to clinical contexts where VOR function is compromised.

## Materials and methods

### Human experiments

Twenty-six healthy participants (7 female; age 25 ± 5 yrs, height 178 ± 6 cm, mass 73 ± 10 kg; mean ± SD) with no self-reported history of neurological disorders participated in this study. The protocol was explained prior to the experiment and all participants gave written informed consent. The experiments conformed to the Declaration of Helsinki and were approved by the Medical Ethics Committee at the Erasmus University Medical Centre. All ethical regulations relevant to human research participants were followed.

### Experimental setup

We conducted four experiments involving human participants to study the influence of voluntary eye closure on the rotational VOR. In Experiment 1, we first examined the attenuating effects of closing the eyes on the VOR in multiple directions by rotating participants with whole-body sinusoidal stimuli in roll, pitch and yaw motion. In Experiment 2, we exposed participants to unpredictable whole-body or head-on-body yaw rotations using: (1) continuous whole-body pseudorandom rotations, (2) whole-body step rotations, and (3) head impulse rotations. In Experiment 3, we tracked the time course of VOR attenuation by having participants repeatedly open and close their eyes while exposed to the continuous whole-body pseudorandom yaw rotations. Finally, in Experiment 4, we examined whether the motor commands to open the eyes could reengage the VOR during unexpected whole-body step rotations despite the eyelids being restrained closed. Participants attempted to open their eyelids against an external obstruction that forced their eyes to remain closed.

Experiments requiring whole-body movements were performed using a motion platform capable of generating rotational and translational motion along three axes (FCS-MOOG, Nieuw-Vennep, The Netherlands; see Fig. 1a). A dedicated control system operating at 100 Hz sent pre-programmed motion signals to the six servo-controlled motors actuating the platform. Participants were restrained to a rigid chair mounted on top of the platform using a four-point harness. Each participant’s head was immobilized using an individually molded dental-impression bite board together with a vacuum pillow placed between their neck and the seatback. Participants sat within field coils that were supported by a polyvinyl chloride frame that could be adjusted in height to ensure that the subject’s eyes were in the center of field. During Experiment 1, we measured movements of the platform using a laser mounted on the platform, which projected on a small photocell (0.8 mm pinhole) fixed in space as described by Goumans et al.^25^. The output voltage of the photocell was used to reconstruct the platform motion offline for VOR gain estimates^25^. During Experiments 2-4, we used a uniaxial gyroscope (ADXRS646, Analog Devices, Wilmington, USA) mounted on the platform to measure yaw angular velocity and estimate VOR gains.

### Data recordings

We recorded eye movements using 3D (Experiment 1) or 2D (Experiments 2–4) scleral search coils (Universal Trading Ventures, Cleveland, USA; and Skalar, Delft, the Netherlands). We used a standard 25 kHz two field coil system (Model EMP3020; Skalar Medical, Delft, the Netherlands) based on the amplitude detection method of Robinson (1963)^40^. We recorded only the left eye in the whole-body movement trials (Experiment 1), while both eyes were recorded in most of the remaining experiments. Prior to coil insertion, the eyes were anaesthetized with a few drops of oxybuprocain (0.4%) in HCl (pH 4.0). All coils were inserted with the wire exiting nasally. During the head impulse rotations (Experiment 2), we measured eye motion in only the left eye as the second coil was used to measure head motion by fixing the coil to an individually molded bite board. During Experiment 3, we also measured eyelid position in the right eye using the magnetic distance measurement technique developed by Koekkoek et al.^41^. This allowed us to track eyelid movement in real-time, ensuring experimental compliance. Briefly, the magnetic sensor was taped to the skin over the maxilla bone underneath the right eye and aligned with a magnet (~0.1 g, 5 × 3 × 1 mm) that was taped to the upper eyelid of the right eye. The voltage signal from the sensor was calibrated for each participant by measuring the actual distance between the sensor and magnet using calipers when the eye was open and closed (Kanon, Tokyo, Japan). Coil data, photocell, magnetic distance and angular rate signals were passed through a 2nd order low-pass filter with a cutoff of 1000 Hz (CyberAmp 380, Axon Instruments, Foster City, USA) prior to digitization with a data acquisition board at 2000 Hz (CED 1401, Cambridge Electronic Design, Cambridge, UK).

### Experimental protocol

Prior to all experiments, we calibrated the coils in two (Experiments 2–4) or three (Experiment 1) dimensions using a gimbal system placed in the center of the magnetic fields. Once the coils were inserted on the eye(s), participants fixated a series of five targets (a central target and four others at 10 degrees left, right, up and down) for ~5 sec each in order to calibrate the horizontal and vertical coil signals and correct for any misalignment of the coils within the magnetic field. The calibration targets were projected onto a translucent screen at a distance of 186 cm. Each participant’s head was oriented comfortably with the Reid’s plane angled ~10° up from the horizontal. The platform center of rotation was aligned with the location of the external meatus and midway between the ears for the whole-body motion trials. Participants were exposed to whole-body rotational motion or head-on-body motion depending on the specific experiment, as explained below. Prior to any of the trials, participants were trained to lightly open and close their eyes to minimize interaction with the search coil leads when forcibly closing their eyes through activity of the orbicularis muscles. In all conditions, participants opened and closed their eyelids to auditory (i.e., a tone or experimenter instructions) and visual cues (i.e., a target extinguishing). The structured timing of eyelid closure and the requirement to follow these cues throughout the different trials required participants to remain attentive to the task throughout the experiments.

*Experiment 1:* Our first experiment examined the effect of eye closure on the VOR during sinusoidal whole-body motion across different directions of motion. Nine participants performed two separate trials: 1) with eyes open in complete darkness, and 2) with eyes closed in complete darkness. During each trial, participants were exposed to 1 Hz sinusoidal rotations around the three cardinal axes (peak velocity of 12.5°/s)—the rostro-caudal axis (yaw), the interaural axis (pitch) and the naso-occipital axis (roll). Along each axis of rotation, the stimuli lasted 14 seconds each with a 2 sec fade-in and fade-out period, providing 10 oscillations for analysis. We expected that eye closure would reduce VOR gains even though visual fixation had been eliminated by complete darkness. A visual target (LED) was briefly presented (~3 s) at the start of each trial when the platform was stationary. During eyes closed trials, participants opened their eyes between each trial when they heard a sound cue and closed them again when the visual target was extinguished. After a random period of 3-4 seconds, the platform motion was initiated. Participants were instructed to fixate the imagined location of the target during the motion after the target had been switched off or they had closed their eyes.

*Experiment 2*: In our second experiment, we used three different movement paradigms to deliver unpredictable whole-body or head-on-body yaw rotations in complete darkness while participants had their eyes either open or closed. These unpredictable body rotations were used to control for the potential predictability of the 1 Hz sinusoidal trials in Experiment 1, which might allow participants to volitionally control eye movements throughout the motion. Each motion paradigm was tested in a different experimental session with a different group of participants.

First, seven participants (of whom one participated in Experiment 1) were exposed to a continuous pseudorandom (i.e., sum-of-sines) whole-body yaw rotation. The pseudorandom signal was comprised of 5 prime harmonics (0.44, 0.92, 1.24, 1.72, and 2.12 Hz) of a common base frequency (0.04 Hz), and had a period of 25 seconds and a peak velocity of 30 °/s. The rotation stimulus lasted 65 seconds and had a 5 s fade-in and fade-out period, ensuring that participants were exposed to two full periods of the stimulus cycle. Second, ten participants (of whom three participated in Experiment 1) were exposed to transient whole-body rotations in the rostro-caudal axis (yaw) with a square wave acceleration signal of 280 °/s^2^ and duration of 125 msec resulting in an angular displacement of 2° and peak velocity of 35 °/s. Rotational transients were repeated 10 times in both directions (leftward and rightward) in a random order and timing between each stimulus was randomized between 4.5 and 7 seconds. Third, five participants (of whom one participated in Experiment 1) underwent head impulse rotations (all center-out), a condition used in clinical evaluations of the VOR that evokes head movement rotation magnitudes commonly observed during daily activities. Participants sat upright looking forward while an experimenter applied transient, high-velocity (up to 150 °/s) head impulses. Head impulses were repeated 10 times in both directions (leftward and rightward) in a random order. Timing between each stimulus was randomized between 4 and 8 seconds. For all three motion conditions, participants followed similar instructions to those provided in Experiment 1. Specifically, an LED target was briefly presented (~2 s) prior to the different movements. During the eyes closed trials, participants closed their eyes when the visual target was extinguished, and when the motion was finished, a sound cue was used to instruct participants to open their eyes in preparation for the next trial. Participants were also instructed to fixate on the imagined location of the target in both conditions.

*Experiment 3:* Here, we evaluated the time course of the observed VOR attenuation in Experiments 1 and 2 when closing the eyes. Seven participants (the same ones that performed the pseudorandom trials in Experiment 2) were exposed to the same continuous pseudorandom whole-body yaw rotation while volitionally opening and closing both eyes according to audio cues given randomly every 4–7 seconds. Participants performed 20 transitions of both opening and closing their eyes over four trials each lasting 75 seconds (i.e., 10 transitions per trial; 5 opening and 5 closing). Here, we expected that the VOR attenuation would occur at the onset of eye closure and be sustained throughout.

*Experiment 4:* Finally, we assessed whether the motor commands to open the eyelids were related to the engagement of the VOR with the eyes open. Ten participants (the same ones that performed the whole-body motion trials in Experiment 2) were exposed to unexpected transient yaw movements (square wave acceleration of 280 °/s^2^ over 125 ms; peak velocity 35 °/s; angular displacement 2°) while they attempted to open both eyes with the lids restricted from opening using medical tape placed over their eyelids. We hypothesized that if the engagement of the VOR is driven by the motor commands to open the lids, then the VOR gain should increase relative to natural eye closure. Under this condition, participants received an audio cue to attempt to open their eyes approximately 3-4 seconds prior to the delivery of the transient whole-body movement. Before the experiments began, participants were trained to attempt to open their eyelids naturally against the eyelid restriction (i.e., using only their palpibrae muscles) and to avoid raising their eyebrows. This was confirmed visually by the experimenter prior to starting the trials and instructions were given throughout the experiment to remind the participants of their task.

### Animal experiments

#### Surgical procedures

Two healthy monkeys (*Macaca mulatta*) were prepared for chronic eye movement experiments. All procedures were approved by the McGill University Animal Care Committee and were in strict compliance with the guidelines of the Canadian Council on Animal Care. We have complied with all relevant ethical regulations for animal use. The surgical preparation followed the procedures described previously^42^. Briefly, a dental acrylic implant was fixed to each animals skull using stainless-steel screws. A stainless-steel post, embedded in the implant, was used to restrain the animal’s head during the experiment to which a uniaxial gyroscope was rigidly attached. Additionally, an 18–19 mm eye coil, consisting of three loops of Teflon-coated stainless-steel wire, was implanted in the right eye behind the conjunctiva. After surgery, cefazolin (25 mg/kg) and buprenorphine (0.01 mg/kg, IM) were administered as postoperative antibiotic and analgesia, respectively. Animals were allowed a minimum recovery period of 2 weeks before recording sessions began.

#### Data acquisition

During the experiment, the animals were head-restrained and comfortably seated in a primate chair mounted on top of a motion platform driven by a servomotor (72402; Kollmorgen, Radford, VA). We measured eye position using the magnetic search coil technique with a 120 cm diameter magnetic field coil system (CNC Engineering, Enfield, CT). Prior to starting each experiment, we calibrated coil signals using a procedure in which animals generated saccadic eye movements to track laser targets at various horizontal and vertical positions (± 5, 10, 15, 20, 25, and 30). We measured head angular velocity using a uniaxial angular gyroscope (Watson Inc., Eau Claire, WI) firmly secured to the animal’s head post. Angular head velocity and eye position signals were low-pass filtered at 250 Hz (8 pole Bessel filter) and sampled at 1000 Hz.

#### Experimental protocol

Monkeys were exposed to a similar continuous pseudorandom whole-body yaw rotation used in Experiments 2 and 3 of our human participant testing, being comprised of five prime harmonics (0.44, 0.92, 1.24, 1.72, and 2.12 Hz) with a common base frequency of 0.04 Hz, a period of 25 seconds, and a peak velocity of 40 °/s. Because the monkeys were not trained to voluntarily close their eyes, the whole-body rotation was repeated several times in order to collect at least 15 instances of voluntary eye closure and opening during rotation per animal. During the periods of movement, voluntary eye closure occurred on an irregular basis, but these episodes were not accompanied by indicators of reduced alertness (i.e., positional eye drift or reduced saccade frequency). Each repetition of the motion signal was separated by 4 s stationary periods. To ensure the monkeys were alert and engaged throughout the recording session, animals received liquid rewards during the short stationary periods. The approximate periods of eye closing and opening were recorded by the experimenter manually pressing a button when the eyes were observed to be closed. The exact timing of eye closure was estimated relative to the manual trigger by identifying the saccadic eye movements that accompany eye closure (see Data analysis). Data from periods of at least 3 s of eye closure and 3 s of eyes open between each closing were included in the analysis. On average, the monkeys closed their eyes for periods of 8.3 ± 11.5 s (monkey A) and 8.8 ± 6.1 s (monkey S).

### Data analysis

#### Human experiments

To assess the attenuation of the vestibular-evoked eye movements across our different conditions, we estimated the gain of the VOR as the ratio between the eye and applied head angular velocity using analyses specific to each motion profile. For sinusoidal trials in Experiment 1, three-dimensional coil signals were first converted into Fick angles and expressed as rotation vectors^43,44^. Misalignment of the coil in the eye relative to the primary magnetic field coils was corrected by three-dimensional counter rotations using the data from the calibration trial when participants were looking at the central target. Eye rotation vectors were then filtered using a 40-point Gaussian window (length of 20 ms) and differentiated using a 5-point central difference algorithm to provide eye angular velocities. Saccades were identified when the variance of the horizontal eye velocity calculated over an 80-point (40 msec) moving window exceeded 50 °/s, and the data during the saccade were removed from further analysis. The start and end times of the saccades were identified manually. Eye velocity signals in the horizontal, vertical, and torsional directions were then fit with a sinusoid having a frequency equal to the platform motion (i.e., 1 Hz). The VOR gain for each direction was then defined as the ratio between the fit peak eye velocity and peak platform velocity^25^, and a total VOR gain was calculated as the vector magnitude of the gains from all three directions.

For the transient motion and head impulse trials in Experiment 2, individual horizontal eye traces were first inspected and removed from further analysis when saccades and blinks were observed during the acceleration phase of the motion disturbance. This occurred on average less than one in every 10 trials. The two-dimensional coil signals were converted to Fick angles, filtered using the same Gaussian window used in Experiment 1 and differentiated to provide eye angular velocities. Angular velocity signals from the gyroscope were filtered using a phaseless sixth-order low-pass Butterworth filter with a cutoff of 15 Hz. Eye velocity signals were then aligned in time to the onset of motion using the gyroscope signal and VOR gains were estimated by performing a linear regression (Eq. 1) between the low-pass filtered (cutoff of 15 Hz) input platform rotation (H’(t)) and the horizontal eye velocity (E’(t)) over the first 100 ms of acceleration. In some participants, closing the eyes led to eye movements that were in the same direction as the applied whole-body motion (see Results). In these cases, VOR gains were set to zero as their linear regressions on eye movement in the same direction as the applied motion produced gains less than zero.

1 $${E}^{{\prime} }\left(t\right)={gain}* H^{\prime} (t)$$

For the head impulse rotations in Experiment 2, we used the coil mounted on the bite board as input head rotation H’(t) instead of a gyroscope signal and estimated the gain over a period of 100 msec. This time window was of sufficient duration to capture the entire period of acceleration applied to the participants. For each motion condition (whole-body and head-on-body rotations), VOR gains were then averaged across all trials as well as across the left and right eye (when both eyes were measured) to provide a single estimate of the VOR gain for each participant.

For the continuous pseudorandom motion trials in Experiments 2 and 3, eye velocities from the two-dimensional coils were processed using the same filter and saccade removal techniques as described for the sinusoidal trials. VOR gains were then estimated using a moving window technique in order to track the time-course of the VOR throughout the experiments. A linear regression between the low-pass filtered (cutoff of 15 Hz) platform rotation (H’(t)) and the horizontal eye velocity (E’(t)) (Eq. 1) was performed over a 300-point (150 ms) moving window (1 ms shifts). In Experiment 2, VOR gains per participant were then estimated from these time-varying signals by taking the average over two periods of the pseudorandom stimulus (i.e., 50 seconds). Finally, a single VOR gain estimate was calculated per participant by averaging across the left and right eye.

In Experiment 3, the linear regression was performed on the separate 6 sec segments of data that were extracted 3 seconds before and after the initiation of eyelid movement when either closing or opening the eyes. Eyelid movement initiation was identified as the instant when the right eyelid sensor velocity exceeded three times the standard deviation from baseline. Baseline eyelid velocity was calculated over a window of 0.5 seconds immediately before the sound cue was given to open or close the eyes. The correct timing of the eyelid movement onset was confirmed visually for every segment of data (see Fig. 3, top traces). Closing and opening of the eye also evoked high velocity horizontal and vertical eye movements consistent with previous reports (see^22^) that were identified by our algorithm as saccades (see Fig. 3, middle traces). Similar to normal saccades, these eye movements induced a substantial distortion in the estimate of VOR gain. We chose, however, to retain these data when estimating the VOR gain throughout the transitions, as removing the data would leave a gap in the VOR gain. We identified this period of eye movement by visually examining the vertical eye velocity traces, which were otherwise stable throughout the trial as the horizontal platform motion did not evoke vertical VOR eye movements. We quantified the timing of these saccadic eye movements (both onset and cessation) relative to the onset of eyelid movement (see arrows in Fig. 3). In each participant and eye, the time-varying VOR gains were averaged across the 20 repetitions of both eye closing and opening. From these mean time-varying signals, we estimated the mean VOR gains over three time periods relative to transitions in eye closure: i) 1–0.5s before, ii) 0.5–1s after, and iii) 2.5–3seconds after. We also characterized the VOR gains 0.5–1 s after eye opening. Due to the transient, tonic and oscillatory nature of the eye movements evoked during eyelid closing, the time-varying VOR gains estimated just after eye closure were occasionally negative (see Results). In these cases, the average gain estimated for each time period was set to zero for further analysis. Finally, for statistical analysis (see below), VOR gains were averaged across the left and right eye to provide a single estimate in these time periods for each participant. Note, for illustrative purposes, we have plotted time-varying gain response from each eye separately to demonstrate their similar behavior (see Results).

#### Animal data

Angular head velocity and eye position signals were digitally filtered with zero phase at 125 Hz using a 51st-order finite-impulse-response filter with a Hamming window. We calculated eye velocity using a first-order backward difference and then we removed saccadic eye movements manually before computing the gain of the VOR. As in humans, eye closure induced saccadic eye movements in both the horizontal and vertical directions. Therefore, based on the observations from our human data, we chose to use properties of the vertical saccade to identify a more precise moment of eye closure as compared to the manual button press from the experimenter. First, we identified the first peak velocity in the vertical eye movements preceding the button press and used this time point to align the separate occurrences of eye closure (see Fig. 4). In addition, similar to the human data, we quantified the timing of this vertical eye movement (both onset and cessation) in the position trace relative to the identified peak in vertical eye velocity (see arrows in Fig. 4). We note that this timing is to provide a better estimate of the moment of eyelid closure but does not reflect the accurate timing of these events that was possible in our human data. From these segments of data, the time-varying VOR gains were then calculated using the technique described for Experiment 3 in human participants (i.e., the moving window technique). We also extracted mean VOR gains before (1–0.5 s), just after (0.5–1 s), and at a later time after (2.5–3 s) eyelid closing for statistical analysis. Mean VOR gains just after eyelid opening (0.5–1 s) were also extracted from the data for comparison.

### Statistics and reproducibility

For statistical analyses, we used SPSS version 25 (IBM Chicago, IL, USA), and the significance level was set at 0.05. For Experiment 1, we first analysed the VOR gains from the sinusoidal trials using a two-way repeated-measures ANOVA with rotation direction and eye condition (eyes open in the dark and eyes closed in the dark) as main factors. Following our hypothesis that eye closure would influence the VOR, we expected gains to decrease during eyes closed conditions. Furthermore, based on previous studies, we expected a main effect of rotation direction, whereby the VOR gain would progressively decrease as the roll component of the applied movement increased. When interactions emerged, we then quantified how much the VOR gains decreased as a result of eye closure in each of the cardinal axes, and compared their relative effect on the VOR using paired Student *t* tests. For Experiment 2, we analysed VOR gains using separate paired Student t-tests to compare eyes open and closed conditions in each motion condition (i.e., whole-body pseudorandom, whole-body transients and head-on-body transients). We used a Holm correction to account for multiple comparisons. Similar to Experiment 1, we expected that eye closure would also decrease VOR gains in all of the unexpected motion conditions. For Experiment 3, we compared the mean VOR gains over the three instances in time (see above) surrounding the initiation of eye closing using a repeated measures ANOVA. A qualitative comparison was made with the animal data. Finally, to determine if the motor commands to open the eyelid increased VOR gains, we compared data from Experiment 4 using a paired Student t-test between eyes closed and when participant attempted to open their eyes against a restriction.

### Reporting summary

Further information on research design is available in the Nature Portfolio Reporting Summary linked to this article.

## Supplementary information

Reporting summary

## Data Availability

The source Matlab data that support the findings of this study and generate the main figure results are available in “Data and code for “Rapid switching of vestibulo-motor pathways through voluntary eyelid closure in primates”” 10.34894/MRYE0Q, DataverseNL^45^. Datasets generated and analyzed during the study are available from the corresponding author upon reasonable request.

## Code availability

The source Matlab code used to generate the main results are available in “Data and code for “Rapid switching of vestibulo-motor pathways through voluntary eyelid closure in primates”” 10.34894/MRYE0Q, DataverseNL^45^.
