# Supplementary material for: Rapid switching of vestibulo-motor pathways through voluntary eyelid closure in primates
Source: Commun Biol. 2025 Nov 20;8:1617. doi: 10.1038/s42003-025-08956-2 (PMC12635057; doi:10.1038/s42003-025-08956-2)
Supplement: Supplementary file 1 — Reporting summary [file 42003_2025_8956_MOESM1_ESM.pdf]

nature portfolio

Corresponding author(s): Patrick A. Forbes

Last updated by author(s): Aug 20, 2025

Reporting Summary

Nature Portfolio wishes to improve the reproducibility of the work that we publish. This form provides structure for consistency and transparency in reporting. For further information on Nature Portfolio policies, see our Editorial Policies and the Editorial Policy Checklist.

Statistics

For all statistical analyses, confirm that the following items are present in the figure legend, table legend, main text, or Methods section.

n/a	Confirmed	
☐	☒	The exact sample size (*n*) for each experimental group/condition, given as a discrete number and unit of measurement
☐	☒	A statement on whether measurements were taken from distinct samples or whether the same sample was measured repeatedly
☐	☒	The statistical test(s) used AND whether they are one- or two-sided<br>*Only common tests should be described solely by name; describe more complex techniques in the Methods section.*
☐	☒	A description of all covariates tested
☐	☒	A description of any assumptions or corrections, such as tests of normality and adjustment for multiple comparisons
☐	☒	A full description of the statistical parameters including central tendency (e.g. means) or other basic estimates (e.g. regression coefficient) AND variation (e.g. standard deviation) or associated estimates of uncertainty (e.g. confidence intervals)
☐	☒	For null hypothesis testing, the test statistic (e.g. F, t, r) with confidence intervals, effect sizes, degrees of freedom and P value noted<br>*Give P values as exact values whenever suitable.*
☒	☐	For Bayesian analysis, information on the choice of priors and Markov chain Monte Carlo settings
☒	☐	For hierarchical and complex designs, identification of the appropriate level for tests and full reporting of outcomes
☒	☐	Estimates of effect sizes (e.g. Cohen's *d*, Pearson's *r*), indicating how they were calculated

Our web collection on statistics for biologists contains articles on many of the points above.

Software and code

Policy information about availability of computer code

Data collection	Custom designed code was used to operate the motion platforms and collect data in the current study.
Data analysis	All data analyses were conducted using MATLAB R2023b.

For manuscripts utilizing custom algorithms or software that are central to the research but not yet described in published literature, software must be made available to editors and reviewers. We strongly encourage code deposition in a community repository (e.g. GitHub). See the Nature Portfolio guidelines for submitting code & software for further information.

Data

Policy information about availability of data

All manuscripts must include a data availability statement. This statement should provide the following information, where applicable:

- Accession codes, unique identifiers, or web links for publicly available datasets
- A description of any restrictions on data availability
- For clinical datasets or third party data, please ensure that the statement adheres to our policy

The datasets generated and analyzed during the study are available from the corresponding author on reasonable request. Data used for generating the plots in the main figures are available in an online repository.

Research involving human participants, their data, or biological material

Policy information about studies with human participants or human data. See also policy information about sex, gender (identity/presentation), and sexual orientation and race, ethnicity and racism.

Reporting on sex and gender	Neither sex nor gender was considered in the study design. Sex was self-reported. A total of 26 volunteers participated in this study (7 females, 19 males).
Reporting on race, ethnicity, or other socially relevant groupings	No race, ethnicity, or other socially relevant variables were included in this work.
Population characteristics	The research sample was composed mainly of undergraduate or graduate students from Erasmus MC. The mean age of participants was 25 (SD: 5) years old.
Recruitment	Participants were recruited by an announcement with the invitation to participate in the experiment which was placed around the Erasmus MC facility.
Ethics oversight	This study was approved by the Medical Research Ethics Committee Erasmus MC (MEC-2016-327)

Note that full information on the approval of the study protocol must also be provided in the manuscript.

Field-specific reporting

Please select the one below that is the best fit for your research. If you are not sure, read the appropriate sections before making your selection.

☒ Life sciences ☐ Behavioural & social sciences ☐ Ecological, evolutionary & environmental sciences

For a reference copy of the document with all sections, see nature.com/documents/nr-reporting-summary-flat.pdf

Life sciences study design

All studies must disclose on these points even when the disclosure is negative.

Sample size	No sample-size calculation was performed. The sample size was chosen based on previous experiments performed in the lab and in line with other studies evaluating human and monkey VORs. See for example:<br>Goumans J, Houben MM, Dits J, van der Steen J. Peaks and troughs of three-dimensional vestibulo-ocular reflex in humans. J Assoc Res Otolaryngol. 2010 Sep;11(3):383-93. doi: 10.1007/s10162-010-0210-y. Epub 2010 Feb 23.<br>Roy JE, Cullen KE. A neural correlate for vestibulo-ocular reflex suppression during voluntary eye-head gaze shifts. Nat Neurosci. 1998 Sep;1(5):404-10. doi: 10.1038/1619.
Data exclusions	There were no data exclusions.
Replication	Several analyzes were performed to ensure the validity of the presented results. Different cohorts of participants were analyzed validating the obtained outcomes.
Randomization	Experimental conditions were randomized specific to each experimental design.
Blinding	Participants were recruited for each experiment but the investigators were not blind to this allocation. Blinding was not necessary for this study as the investigators awareness of group allocation could not influence the results.

Reporting for specific materials, systems and methods

We require information from authors about some types of materials, experimental systems and methods used in many studies. Here, indicate whether each material, system or method listed is relevant to your study. If you are not sure if a list item applies to your research, read the appropriate section before selecting a response.

Materials & experimental systems

n/a	Involved in the study
☒	☐ Antibodies
☒	☐ Eukaryotic cell lines
☒	☐ Palaeontology and archaeology
☐	☒ Animals and other organisms
☒	☐ Clinical data
☒	☐ Dual use research of concern
☒	☐ Plants

Methods

n/a	Involved in the study
☒	☐ ChIP-seq
☒	☐ Flow cytometry
☒	☐ MRI-based neuroimaging

Animals and other research organisms

Policy information about studies involving animals; ARRIVE guidelines recommended for reporting animal research, and Sex and Gender in Research

Laboratory animals	Two healthy monkeys (Macaca mulatta) were prepared for chronic eye movement experiments.
Wild animals	The study did not involve wild animals
Reporting on sex	Neither sex nor gender was considered in the study design.
Field-collected samples	The study did not involve samples collected from the field.
Ethics oversight	The experimental protocols were approved by the McGill University Animal Care Committee and were in compliance with the guidelines of the Canadian Council on Animal Care.

Note that full information on the approval of the study protocol must also be provided in the manuscript.

Plants

Seed stocks	Not applicable
Novel plant genotypes	Not applicable
Authentication	Not applicable
